# Case Report: Roxadustat in Combination With Rituximab Was Used to Treat EPO-Induced Pure Red Cell Aplasia

**DOI:** 10.3389/fneph.2022.847847

**Published:** 2022-03-24

**Authors:** Xiaoe You, Baochun Guo, Zhen Wang, Hualin Ma, Ru Zhou, Lixia Liu, Xinzhou Zhang

**Affiliations:** ^1^ The Second Clinical Medical College, Jinan University (Shenzhen People’s Hospital), Shenzhen, China; ^2^ Department of Nephrology, Shenzhen Peoples Hospital (The Second Clinical Medical College, Jinan University; The First Affiliated Hospital, Southern University of Science and Technology), Shenzhen, China; ^3^ Shenzhen key Laboratory of Kidney Diseases, Shenzhen People’s Hospital (The Second Clinical Medical College, Jinan University; The First Affiliated Hospital, Southern University of Science and Technology), Shenzhen, China

**Keywords:** anti-EPO antibodies, erythropoietin, pure red cell aplasia, roxadustat (FG-4592), rituximab, case report

## Abstract

Recombinant human erythropoietin (rHuEPO) is a drug given to patients who have low hemoglobin related to chronic kidney disease or other anemia-related diseases. Some patients who receive rHuEPO repeatedly develop anti-rHuEPO-neutralizing antibodies, leading to the occurrence of pure red cell aplasia (PRCA). PRCA associated with rHuEPO includes severe rHuEPO resistance, blood transfusion dependence, high serum ferritin, severe reticulocytopenia, and presence of anti-rHuEPO antibody. However, the optimal treatment of erythropoietin (EPO)-induced PRCA is unclear. Therapeutic options against it remain a major clinical challenge. Herein we report on 2 male patients with PRCA during rHuEPO treatment, who received a combination therapy of roxadustat plus rituximab but had completely different clinical outcomes. The results obtained in this study show that roxadustat in combination with rituximab could be one of the treatment options for EPO-induced PRCA, but the treatment efficacy can vary from one individual to another. Additionally, we recommend starting reticulocyte monitoring and immunosuppressive agent therapy as early as possible to shorten the course of the disease and improve the outcomes of the patients.

## Introduction

Recombinant human erythropoietin (rHuEPO) is a drug given to patients who have low hemoglobin related to chronic kidney disease or other anemia-related diseases. However, some patients who receive rHuEPO repeatedly develop anti-rHuEPO neutralizing antibodies, leading to the occurrence of pure red cell aplasia (PRCA) (1). The possible reasons include the subcutaneous administration route, formulation, type, and the preparation of the epoetin itself (2). PRCA associated with rHuEPO includes severe rHuEPO resistance, blood transfusion dependence, high serum ferritin, severe reticulocytopenia, and presence of anti-rHuEPO antibody (2). Thrombocytopenia is also one of its manifestations (1). The result of a bone marrow aspirate suggests very low erythroid progenitor cells while the other blood cell parameters are normal (3). The general treatment plan is to stop erythropoietin and use corticosteroids, cyclophosphamide, cyclosporin A, intravenous immunoglobulin, kidney transplantation, *etc.* (4). Peginesatide has also been reported to be effective (5), but the optimal treatment of anti-erythropoietin antibody-mediated PRCA is unclear. Therapeutic options against PRCA remain a major clinical challenge.

Roxadustat and rituximab, either drug alone, have also been reported to be effective against erythropoietin (EPO)-induced PRCA. Roxadustat is a hypoxia-inducible factor prolyl hydroxylase inhibitor that regulates the expression of EPO and hepcidin as well as others, thus promoting erythropoiesis at multiple levels (6, 7). Multiple reports have implicated that roxadustat could effectively treat EPO-induced PRCA after an immunosuppressive treatment (8–11). It has been even suggested that roxadustat still shows good efficacy without immunosuppressive therapy (12). Rituximab is a genetically engineered monoclonal antibody directed against CD20 antigen, is seen in a certain subset of B-cells, and causes significant B-cell depletion. Rituximab has shown good efficacy in many autoimmune hematologic disorders together with low or absent toxicity (13, 14) and has already been administered in other cases of PRCA (15, 16). Cases of epoetin-related PRCA treated with rituximab which succeeded or failed had been reported (17, 18). Then, how is the efficacy in the combination therapy of roxadustat and rituximab? Is this combination therapy superior to monotherapy for treating EPO-induced PRCA?

Therefore, the present report is the first to describe 2 cases of PRCA related to rHuEPO whose hemoglobin had varying outcomes after a combination therapy of roxadustat and rituximab.

## Case Report

### Case 1

**Figure 1:**
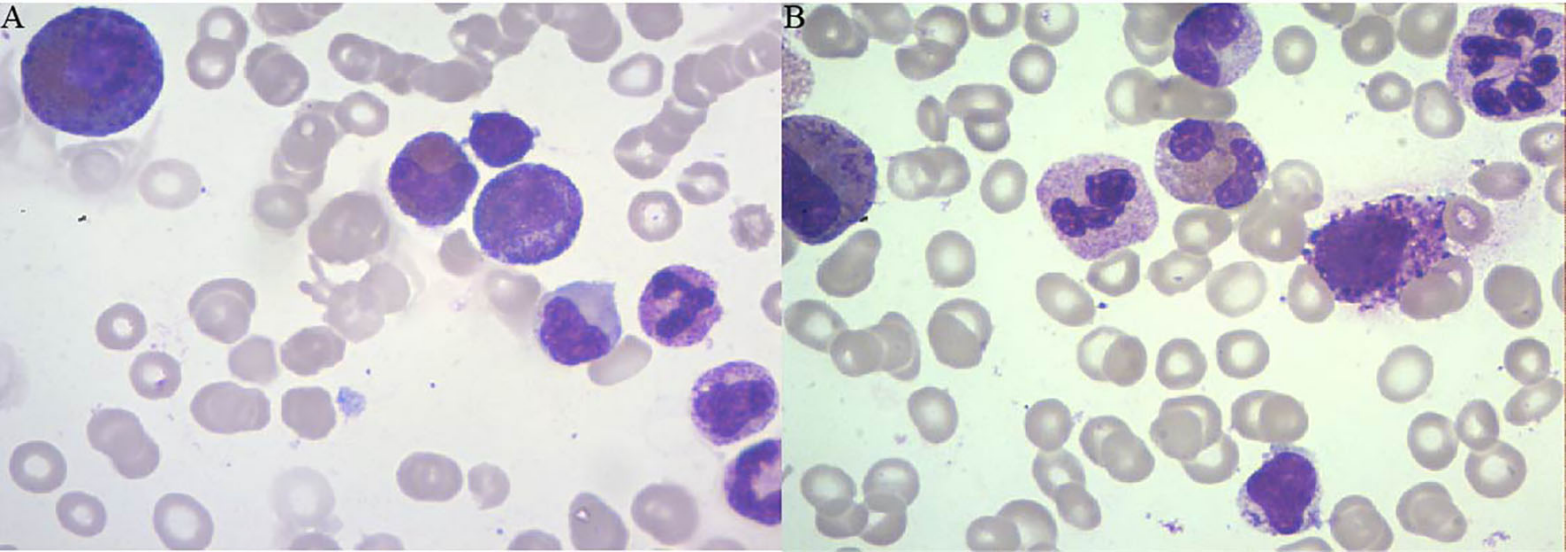
The results of the bone marrow biopsy of patient 1. **(A)** Bone marrow biopsy (June 4, 2019) showing the absence of erythroblasts and normal myeloid series and platelet precursors. **(B)** Bone marrow biopsy (December 23, 2020) showing hypoplasia of the erythroid line.

**Figure 2:**
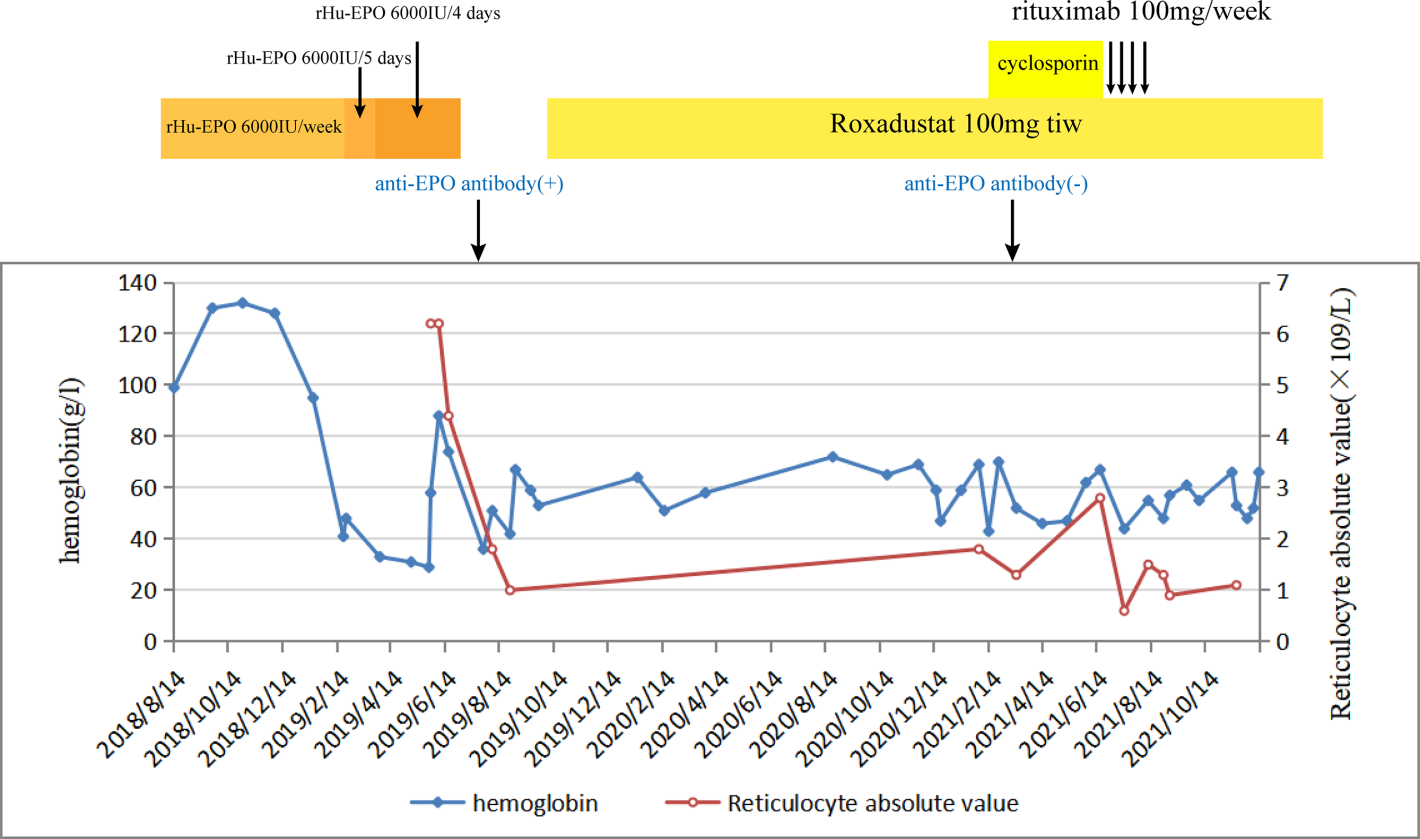
The change in hemoglobin and reticulocyte absolute values during the whole course of the disease of patient 1.

**Figure 3:**
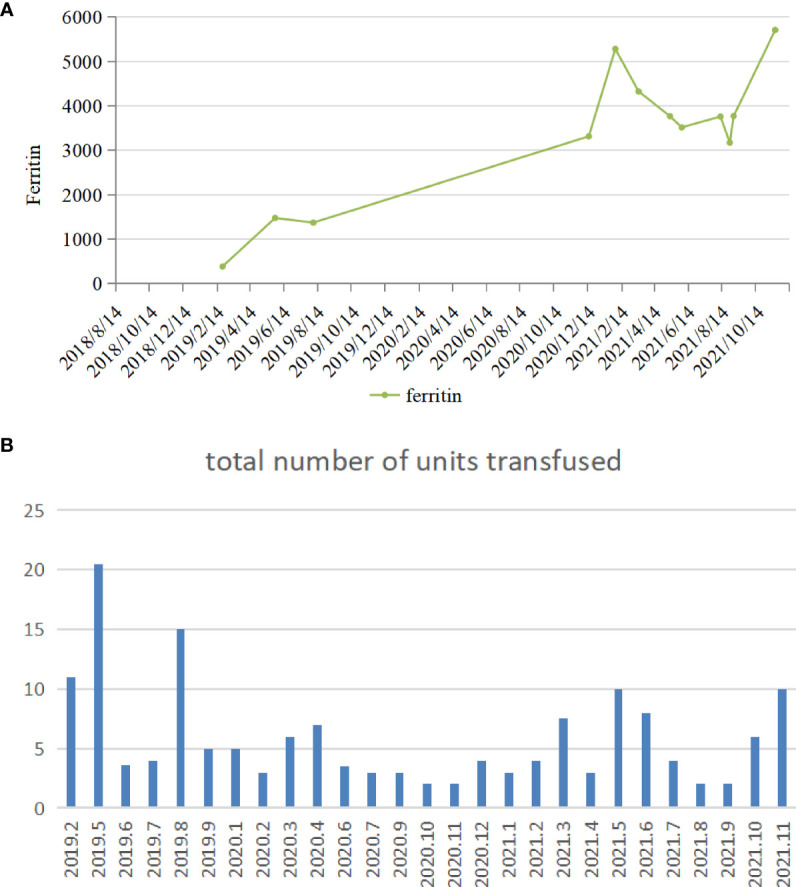
**(A)** The variation tendency of ferritin of patient 1. **(B)** The number of transfusions in patient 1.

A 54-year-old man was diagnosed with chronic kidney disease (CKD) for the first time in October 2017. The possible reason for CKD was chronic glomerulonephritis, but a renal biopsy was not performed. He had prior viral hepatitis B with normal hepatic function and nucleic acid quantification. In August 2018, renal anemia was diagnosed in this patient, and erythropoietin-stimulating agents (ESAs) were used once a week [epoetin-α, 6,000 international units (IU); Shenzhen, China], with his hemoglobin stable at above 120 g/L. In February 2019, the patient’s hemoglobin suddenly dropped to 48 g/L. As the gastroscopy result suggested gastrointestinal bleeding, we attributed the decrease in hemoglobin to gastrointestinal bleeding. He underwent endoscopic hemostatic therapy and had an increased dose of erythropoietin. However, the hemoglobin concentration was monitored to fluctuate between 31 and 36 g/L. A transient rise in hemoglobin level was achieved with the infusion of a large amount of red suspension, but there was a rapid decrease. The results of a re-examination showed a negative fecal occult blood. The recurrent drop in hemoglobin and transfusion dependency could not be explained by gastrointestinal bleeding. In May 2019, the patient was re-hospitalized with severe anemia. This time, we noted that his reticulocyte absolute value was markedly decreased. Therefore, a bone marrow puncture was performed, and the result showed severe hypoplasia of the erythroid line with hyperplasia of the granulocytic and megakaryocytic lines, normal ratio of blast cells, and no morphologic dysplasia (**Figure 1A**). Anti-DNA anti-body, anti-nuclear anti-body, parvovirus B19 DNA, and tumor biomarker were not detected in the blood. Computed tomography of the chest and abdomen found nothing abnormal. The anti-EPO antibody was checked by ELISA and turned out to be positive (**Supplementary File S1**). Based on the above-mentioned evidence, the final diagnosis of anti-EPO antibody-induced PRCA was established. Therefore, the ESAs were suspended, and roxadustat (150 mg, three times a week) was started to be used in September 2019 (the patient refused immunosuppressive agents). The patient also started to receive hemodialysis. Regrettably, no significant improvements were obtained after 1 year of treatment (**Figure 2**). Thus, we performed bone marrow biopsy and anti-EPO antibody re-review. The bone marrow aspirate still showed hypoplasia of the erythroid line (**Figure 1B**), but the anti-erythropoietin antibody was converted to negative. These results may indicate that the patient achieved partial immunological recovery from EPO-induced PRCA. Later, we started to use cyclosporin A but had discontinued it due to side effects. In May 2021, we began a combined treatment with rituximab (4 cycles of 100 mg/week) and roxadustat (150 mg, three times a week). So far, the patient has stayed on the combination therapy for 8 months, but the patient’s anemia has not improved. Frequent blood transfusions are still needed (**Figure 3A**), and the ferritin levels were significantly increased (**Figure 3B**).

### Case 2

**Figure 4:**
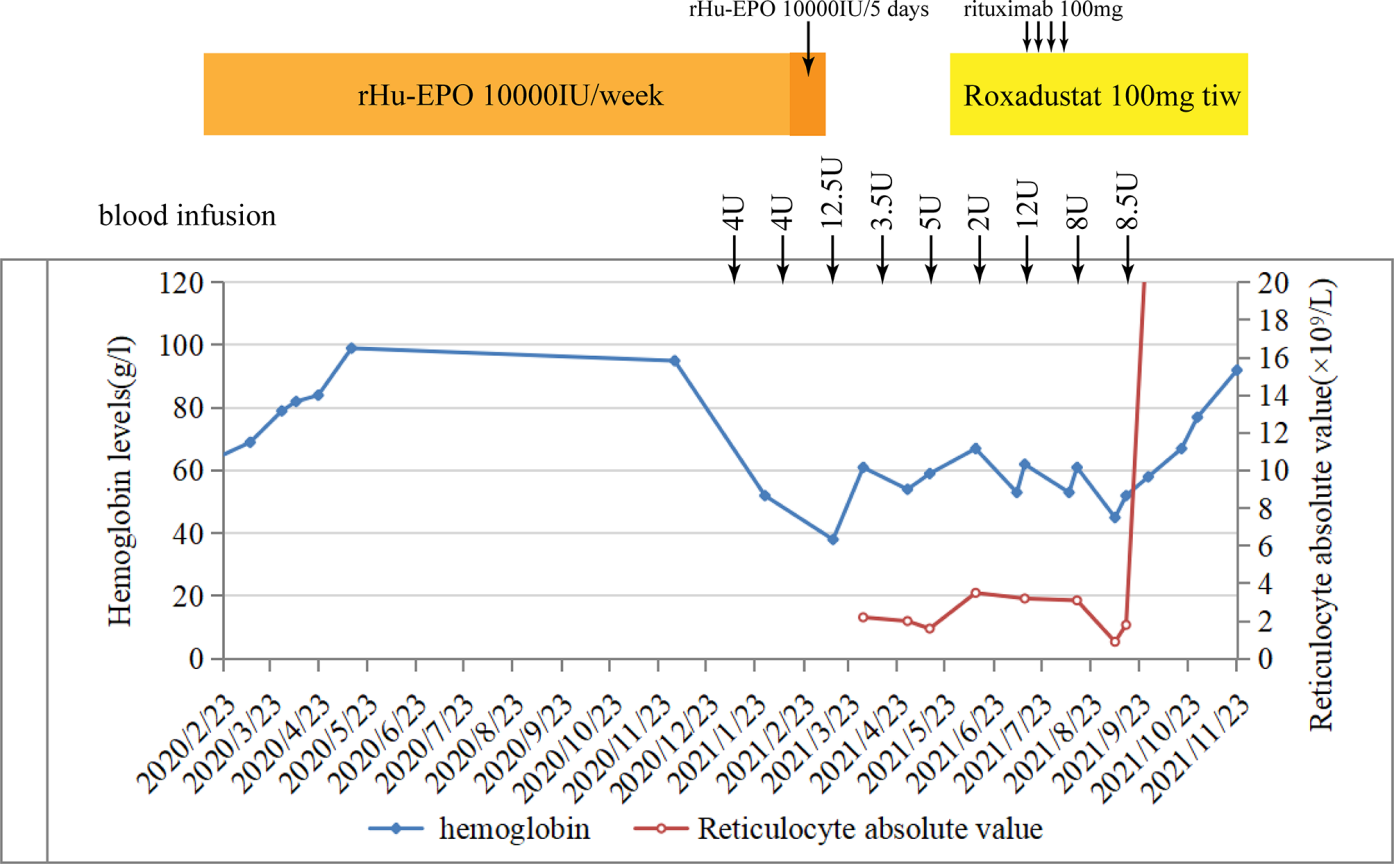
Hemoglobin and reticulocyte absolute values in patient 1 over time and their relation to treatment.

A 72-year-old man was diagnosed with chronic renal failure 5 years earlier, the etiology is hyperuricemic nephropathy. In March 2020, his hemoglobin (Hb) concentration decreased to 63 g/L, and he was diagnosed with renal anemia. He began to be administered with rHuEPO (epoetin-α, 10,000 IU; Shenzhen, China) subcutaneously once a week. His anemia improved substantially, and his Hb level increased to about 100 g/L for 10 months. During that time, his serum creatinine level reached 631 μmol/L, and he received hemodialysis. In January 2021, his Hb suddenly fell to 58 g/L without evidence of bleeding, infection, and hemolysis. In response, his rHuEPO was increased to 10,000 units once every 5 days. However, it turns out that it does not improve the results. In the following 5 months, he was admitted to the outpatient clinic weekly for transfusion of red blood cells, his anemia improved temporarily after transfusion, and then the levels of Hb began to decline rapidly to below 40 g/L. In April 2021, the routine blood examination showed that the Hb concentration was 63 g/L, and the reticulocyte count was 2.2 × 10^9^/L in this patient. Therefore, there was clinical suspicion of ESA-induced PRCA. The ESAs were discontinued, and the use of roxadustat 3 times a week was started. However, a difference in hemoglobin concentration was not seen at subsequent assessments. In July 2021, the bone marrow aspirate showed severe hypoplasia of the erythroid line with hyperplasia of the granulocytic and megakaryocytic lines, normal ratio of blast cells, and no morphologic dysplasia. In this case, there was no evidence of other causes of PRCA* *such as thymoma, hemolysis, lymphoproliferative diseases, infection, or serious malnutrition, but the anti-EPO antibody was checked by ELISA and turned out to be negative (unfortunately, we did not perform a result re-review or the antibody was not measured earlier). Based on proposed case definitions of suspected or proven anti-EPO antibody-induced PRCA in patients treated with epoetin, it can be defined as a suspected case with bone marrow-confirmed PRCA (1). In August 2021, considering the patient’s advanced age, weakened immune system, and obvious side effects of using cyclosporin A or hormones, we began a combined treatment of rituximab (4 cycles of 100 mg/week) and roxadustat (100 mg, three times a week). With the treatment, his Hb subsequently improved to above 7 g/dl for 3 months without blood transfusion, and the reticulocyte absolute value was elevated to 103 × 10^9^/L (**Figure 4**). We did not repeat the bone marrow study since there was clear clinical evidence to prove the stable recovery of the disease. The above-mentioned data indicated that the combination therapy of roxadustat and rituximab was effective in treating PRCA for this patient.

## Discussion

In this article, 2 patients developed worsening anemia following ESA treatment after 6 and 10 months despite a continued therapy with epoetin at the same or even increased doses. Both laboratory workup and bone marrow biopsy showed severe hypoplasia of the erythroid line. Additionally, the anti-EPO antibody was positive in patient 1. Although the anti-EPO antibody was negative in patient 2, there was a high clinical suspicion of erythropoietin-induced PRCA. Both patients have been treated with roxadustat alone at the outset, but the clinical outcome was poor. Because of the side effects of cyclosporin A and the hormones, a combination therapy of roxadustat and rituximab was used. However, different clinical outcomes were observed in the 2 patients.

No significant clinical improvement was observed in patient 1 who received roxadustat, cyclosporin A, and rituximab successively after a diagnosis of EPO-induced PRCA; he remained transfusion dependent. Roxadustat alone has limited effectiveness in the case of ESA-induced PRCA. The reason is possibly caused by the fact that the anti-EPO antibodies were not only against exogenous but also endogenous EPO (2). After 1 year of treatment with roxadustat, although there was no discernible recovery, a re-inspection showed that the anti-erythropoietin antibody converted to negative. This might be because roxadustat can modulate immunity by stabilizing hypoxia-inducible factor (HIF) (19, 20), or endogenous EPO induced by roxadustat neutralized the anti-EPO antibodies but did not boost the production of new antibodies because of lesser immunogenicity than rHuEPO (9). However, the patient’s condition is still not improved in cases of antibody seroconversion, and immunosuppressive agents were used correctly. On the one hand, it is reasonable to question the accuracy of the results of the serum antibodies. Different methods may be helpful. On the other hand, unfortunately, we missed to measure the reticulocyte count in patient 1 during the early stage, which was a regrettable oversight, leading to a delay in this patient’s PRCA diagnosis. At the same time, the long course of the disease and frequent transfusion are also important contributors. Transfusion-dependent patients develop secondary iron overload. As can be seen in **Figure 3A**, this patient had strongly elevated ferritin concentrations. High ferritin levels can be an important indicator of poor prognosis in PRCA. This was a further reminder that enhanced reticulocyte surveillance may help to discover PRCA earlier, and an early combined therapy with aggressive immunosuppressants may help shorten the course of the disease and improve the outcomes in ESA-induced PRCA patients.

Although the anti-EPO antibody was negative in patient 2, it was a case with a high clinical suspicion of erythropoietin-induced PRCA. This reminds us that we could not rely on serum anti-EPO antibodies alone to suspect EPO-induced PRCA. After the diagnosis of ESA-induced PRCA, patient 2 discontinued the ESA for 3 months without taking any oral drug. An unimproved condition indicated that few hematologic recoveries were achieved without immunosuppressive treatment or renal transplantation. Studies indicated that hematological recovery was sustained only in 2% of the patients after withdrawal of the drug, and the recovery rate of patients with immunosuppressive therapy could reach the target of >52% (4, 21). Learning from the experience with patient 1, we started a treatment with rituximab immediately once we found out that roxadustat alone was not effective (no recovery was observed in hemoglobin level and reticulocyte count). Surprisingly, the patient showed a favorable therapeutic response to the combination therapy of roxadustat and rituximab. With the treatment, his hemoglobin level and reticulocyte count rose dramatically, accompanied with a high level of serum EPO. We think that sequential B-cell response abrogation obtained by rituximab, immunomodulatory of HIF, and the change in the administration route may have contributed to the effective treatment and the lower degree of immunogenicity. To avoid symptom recurrence, we did not select to repurpose EPO but continued to use roxadustat. So far, the patient was uneventful and had no relapses.

Why did the 2 patients with EPO-induced PRCA responded so differently to the combination therapy of roxadustat and rituximab? By analyzing the clinical characteristics and courses, we think that there are several reasons. Firstly, the disease duration was significantly longer in patient 1 compared to patient 2, likewise with either diagnosis or treatment. As we have mentioned above, in case 1, we might have underestimated the importance to monitor the level of reticulocytes during the early stage; this can lead to a delay in diagnosis. Simultaneously, the treatment plan is not adjusted timely when we found out that the treatment effect of roxadustat is not good. This might indirectly contribute to a greater number of red blood cell transfusions than in patient 2. Secondly, we observed that patient 1 had a more prominent response to EPO treatment at the beginning than patient 2. This might lead to an increased number of antibodies. As indicated in the antibody testing results, the result of anti-EPO antibody testing was negative for patient 2, which may represent a weaker immune response. It may illustrate that the combination therapy may be more effective in patients with a mild disease. However, these ideas are conjectures at present. It is possible that there may be other underlying causes present which require further investigation.

Overall, the level of hemoglobin and reticulocytes should be monitored in patients who have used ESAs for a long time. Once ESA-induced PRCA is suspected, ESAs should be discontinued, and anti-EPO antibody test and bone marrow biopsy should be performed immediately. There are multiple clinical options for the treatment of this disease. The curative effect of roxadustat alone should be further studied. The combination therapy of roxadustat and rituximab may be one of the underlying options for the treatment of ESA-induced PRCA if the alternate monotherapy of roxadustat or another treatment is ineffective and immunosuppressive agents are not tolerated or are contraindicated, but more clinical trials are needed to confirm the efficacy of this regimen. Significantly, during the course of actual drug treatment, care should be taken to balance such potential benefits with the risk of possible side effects. Additionally, starting an immunosuppressive agent therapy as early as possible may help shorten the course of the disease and improve the outcomes of the patients, which need further study.

## Data Availability Statement

The original contributions presented in the study are included in the article/**Supplementary Material**. Further inquiries can be directed to the corresponding author.

## Ethics Statement

Written informed consent was obtained from the individual for the publication of any potentially identifiable images or data included in this article.

## Author Contributions

XY designed the research and wrote the article. BG conceived the idea of the case report. ZW and LL drew the figure. HM, RZ, and XZ revised the article. All authors reviewed the final version of the manuscript and agreed to its submission.

## Funding

This work was supported by the Shenzhen Fund for Guangdong Provincial High-Level Clinical Key Specialties (no. SZGSP001) and the Shenzhen Key Laboratory of Kidney Diseases (ZDSYS201504301616234).

## Conflict of Interest

The authors declare that the research was conducted in the absence of any commercial or financial relationships that could be construed as a potential conflict of interest.

## Publisher’s Note

All claims expressed in this article are solely those of the authors and do not necessarily represent those of their affiliated organizations, or those of the publisher, the editors and the reviewers. Any product that may be evaluated in this article, or claim that may be made by its manufacturer, is not guaranteed or endorsed by the publisher.
