# Supplementary material for: Case Report: Roxadustat in Combination With Rituximab Was Used to Treat EPO-Induced Pure Red Cell Aplasia
Source: Front Nephrol. 2022 Mar 24;2:847847. doi: 10.3389/fneph.2022.847847 (PMC10479623; doi:10.3389/fneph.2022.847847)
Supplement: Supplementary file 1 [file Presentation_1.pdf]

The method of anti-EPO antibody estimation

1. Binding antibody detection

The known rhEPO protein is bound to the solid phase carrier, and then the sample to be tested and the normal human serum control are diluted into different concentrations and added to the coated plate to form an antigen-antibody complex. Then, horseradish peroxidase-labeled goat anti-huma IgG was added to form an enzyme-anti-EPO antibody complex, wash off unreacted substances, add substrate, A450 was measured with a microplate reader, the sample to be tested with same dilution was compared with normal human serum control.

2. Antibody Neutralizing Activity Detection

The basis for the determination of rhEPO neutralizing antibodies is that rhEPO neutralizing antibodies can inhibit the stimulation proliferation effect of rhEPO on dependent cell line UT7/EPO. The serum of patients with positive rhEPO antibody was incubated with rhEPO at 37°C and then added to UT7/EPO cells for culture. The standard curve was compared and the recovery rate was calculated. If the curve deviates seriously and the recovery rate is less than the set value, it can be preliminarily determined that the biological activity of rhEPO is inhibited. Antibody neutralization was defined as inhibition of the biological activity of rhEPO accompanied by clinically significant inhibition of erythropoiesis.
